# Does Total Arterial Revascularisation Confer a Survival Advantage in Moderate Left Ventricular Dysfunction? A Retrospective Cohort Study of 1866 Patients

**DOI:** 10.3390/jcdd13060278

**Published:** 2026-06-19

**Authors:** Albaraa Al-Holy, Nandor Marczin, Sunil K. Bhudia, Shahzad G. Raja

**Affiliations:** 1 Department of Cardiac Surgery, Harefield Hospital, London UB9 6JH, UK; albaraa.alholy1@nhs.net (A.A.-H.); s.bhudia@nhs.net (S.K.B.); 2 Department of Anaesthesia and Intensive Care, Harefield Hospital, London UB9 6JH, UK; nandor.marczin@nhs.net

**Keywords:** coronary artery bypass, left ventricular dysfunction, myocardial revascularisation, survival analysis, total arterial grafting

## Abstract

Objectives: The optimal conduit strategy for coronary artery bypass grafting (CABG) in patients with moderate left ventricular dysfunction (LVEF 30–49%) remains debated. While total arterial grafting (TAG) has shown benefits in broader populations, its role in this higher-risk subgroup is unclear. This study aimed to compare short-term outcomes and long-term survival between single arterial grafting (SAG) and TAG in patients with moderate LV dysfunction undergoing CABG. Methods: A retrospective analysis of 1866 patients was performed, with 640 patients matched using propensity scores (320 SAG vs. 320 TAG). Preoperative, intraoperative, and postoperative variables were assessed. Survival was evaluated using Kaplan–Meier analysis and Cox regression. Results: Matched cohorts were well balanced across baseline characteristics. Long-term survival at 10 and 15 years was numerically higher in the TAG group (85.8% and 79.7%) compared to SAG (81.7% and 74.2%), though not statistically significant (log-rank *p* = 0.862). Multivariate Cox regression identified age (HR 1.045, *p* < 0.001), NYHA class (NYHA III HR 0.610, *p* = 0.003), previous cardiac surgery (HR 0.501, *p* = 0.006), and off-pump CABG (HR 1.521, *p* < 0.001) as independent predictors of mortality. Grafting strategy (TAG vs. SAG) was not independently associated with long-term mortality (HR 1.005, *p* = 0.966). Conclusion: TAG is safe and feasible in patients with moderate LV dysfunction undergoing isolated CABG, with comparable short-term outcomes. Although unadjusted analyses suggested improved long-term survival, this difference was not observed after propensity matching or multivariable adjustment, and grafting strategy was not independently associated with mortality.

## 1. Introduction

Patients with moderate left ventricular (LV) dysfunction (left ventricular ejection fraction [LVEF] 30–49%) represent a clinically important subgroup undergoing coronary artery bypass grafting (CABG). This population carries higher perioperative and long-term risk than patients with preserved LV function, yet is distinct from those with severe dysfunction, in whom operative mortality and adverse events are substantially elevated [1,2,3]. Optimising conduit strategy in this intermediate-risk group remains an area of active investigation.

The choice between single arterial grafting (SAG) and total arterial grafting (TAG) continues to generate debate. While arterial conduits—particularly bilateral internal mammary arteries—have demonstrated superior long-term patency and survival in general CABG populations [4,5,6], evidence in patients with LV dysfunction is less consistent. Early studies suggested that TAG is feasible and safe in impaired LV function [1], and more recent analyses have shown that multiarterial grafting confers a survival advantage across the spectrum of LVEF impairment [7]. However, the specific impact of TAG in patients with moderate LV dysfunction remains poorly defined.

This study aimed to evaluate whether TAG offers superior short-term outcomes and long-term survival compared with SAG in patients with moderate LV dysfunction undergoing isolated CABG. Secondary objectives included identifying independent predictors of long-term mortality and assessing the safety profile of TAG in this population.

## 2. Methods

### 2.1. Study Design and Population

**Figure 1:**
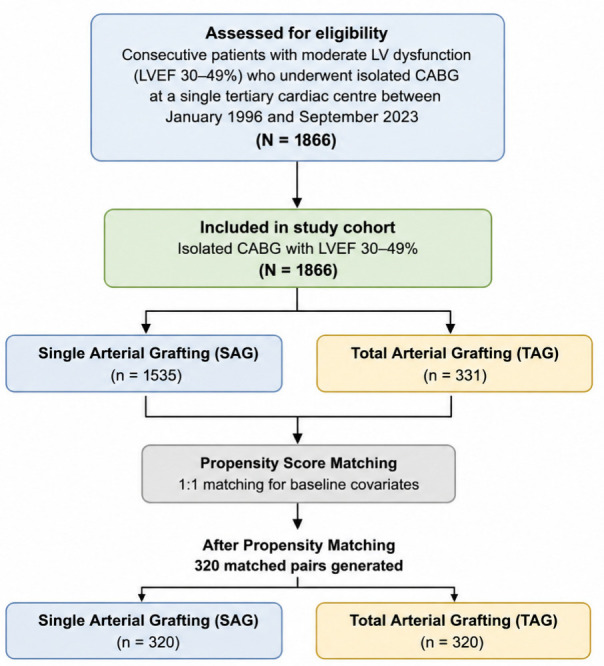
Study flow diagram illustrating patient selection, allocation according to conduit strategy, and propensity score matching.

This retrospective cohort study included 1866 consecutive patients with moderate left ventricular dysfunction, defined as a left ventricular ejection fraction between 30% and 49%, who underwent isolated CABG at a single tertiary cardiac centre between January 1996 and September 2023. Patients were categorised according to conduit strategy into SAG and TAG groups. The process of patient selection and cohort derivation is summarised in Figure 1.

SAG, comprising 1535 patients, was defined as the use of a single arterial conduit, most commonly the left internal mammary artery, supplemented by one or more saphenous vein grafts. TAG, comprising 331 patients, was defined as revascularisation performed exclusively with arterial conduits, with no use of any saphenous vein grafts.

Patients who received two or more arterial grafts in combination with any supplemental vein grafts were excluded to ensure strict separation between arterial-only and mixed-conduit strategies. Accordingly, TAG group consisted solely of patients treated with arterial conduits alone.

Patients undergoing concomitant cardiac procedures or presenting with a left ventricular ejection fraction below 30% or above 50% were excluded. All included cases had complete clinical, operative, and follow-up data and were analysed on a complete-case basis. Ethical approval was obtained from the institutional audit committee, which granted a waiver of individual informed consent due to the retrospective nature of the study and the use of anonymised data under reference QS-SR-04/2024. All procedures were conducted in accordance with the principles of the Declaration of Helsinki.

### 2.2. Data Collection

Data were extracted from a prospectively maintained institutional cardiac surgery database. Variables included demographic characteristics, comorbidities, coronary anatomy, operative details, postoperative complications, and long-term survival. Arterial conduits were defined as internal mammary, radial, or gastroepiploic arteries. TAG was defined as exclusive use of arterial conduits, while SAG referred to the use of a single arterial graft supplemented by saphenous vein grafts. The primary endpoint was long-term all-cause mortality; secondary endpoints included 30-day mortality and major postoperative complications.

### 2.3. Interventions

All patients underwent isolated CABG using standard surgical techniques. Off-pump CABG (OPCAB) or on-pump CABG was performed at the discretion of the operating surgeon. Conduit selection, configuration, and target vessel assignment were based on coronary anatomy, conduit quality, and surgeon preference. During the study period, conduit strategy was determined at the discretion of the operating surgeon and reflected individual clinical judgement. Factors influencing the choice between SAG and TAG included coronary anatomy, target-vessel characteristics, conduit quality and availability, patient age, comorbidity burden, anticipated operative risk, and surgeon experience with arterial revascularisation techniques. Institutional practice evolved over time, with progressively greater adoption of multiarterial and total arterial grafting strategies in selected patients as supporting evidence and surgical expertise increased. TAG strategies included combinations of left and right internal mammary arteries and radial artery grafts.

### 2.4. Statistical Analysis

Continuous variables were assessed for normality using the Shapiro–Wilk test. Normally distributed variables were summarised as mean ± standard deviation and compared using independent-samples *t*-tests; non-normally distributed variables were compared using the Mann–Whitney U test. Categorical variables were compared using χ^2^ or Fisher’s exact test.

To reduce treatment-selection bias, a propensity score was generated using multivariable logistic regression incorporating all baseline variables presented in Table 1. The propensity score model demonstrated good discrimination, with a c-statistic of 0.75. One-to-one nearest-neighbour matching without replacement was then performed using a caliper width of 0.10 of the standard deviation of the logit of the propensity score. Matching was performed on the logit of the propensity score to ensure optimal balance across the covariate distribution.

Covariate balance before and after matching was assessed using standardised mean differences (SMDs), with an SMD of <0.10 indicating adequate balance. In addition, visual inspection of propensity score distributions before and after matching confirmed substantial overlap between groups and demonstrated appropriate achievement of common support. These analyses collectively demonstrated improved baseline comparability following matching.

Of the total cohort, 640 patients were successfully matched, comprising 320 TAG and 320 SAG patients. Although 1535 SAG patients were available in the original cohort, matching was constrained by the available overlap in propensity score distributions. All 331 TAG patients were considered for matching, of whom 320 were successfully matched; the remaining 11 TAG patients could not be matched due to propensity scores falling outside the region of common support, precluding identification of suitable matches within the predefined caliper.

To account for potential confounding related to temporal changes in surgical practice, perioperative management, and secondary prevention over the 1996–2023 study period, calendar time (year of surgery) was incorporated as a continuous covariate in the propensity score model. In addition, sensitivity analyses were performed to assess the robustness of the primary findings. These included (i) a multivariable Cox proportional hazards model adjusting for year of surgery, and (ii) an era-stratified analysis dividing the cohort into early (1996–2004), mid (2005–2014), and late (2015–2023) operative periods. The association between conduit strategy and long-term mortality was reassessed within each era and across adjusted models.

Survival was analysed using Kaplan–Meier methods, with differences assessed using the log-rank test. Cox proportional hazards regression was used to identify predictors of long-term mortality. Variables with *p* < 0.10 in univariable analysis were entered into a multivariable model. Proportional hazards assumptions were evaluated using Schoenfeld residuals. Hazard ratios (HRs) with 95% confidence intervals (CIs) were reported. Statistical significance was defined as *p* < 0.05. All statistical analyses were performed using IBM SPSS Statistics (Version 29.0; IBM Corp., Armonk, NY, USA).

## 3. Results

### 3.1. Preoperative Demographics

**Figure 2:**
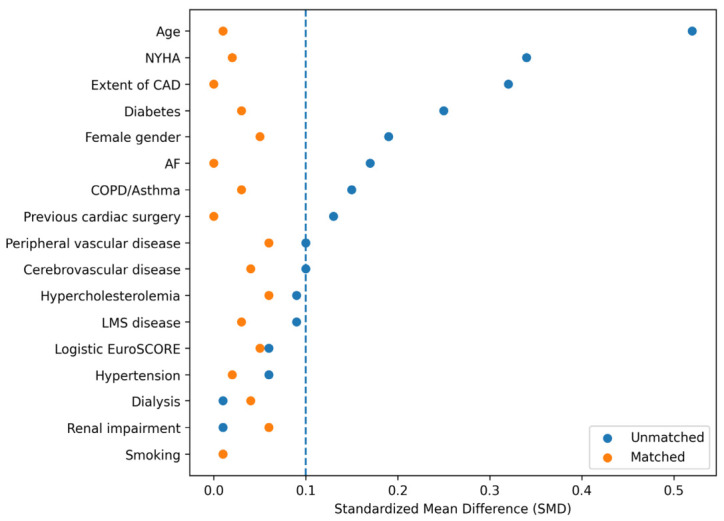
Love plot demonstrating covariate balance before and after propensity score matching.

Baseline characteristics for the unmatched and matched cohorts are presented in Table 1. In the unmatched population, several variables differed significantly between the SAG and TAG groups. TAG patients were younger than SAG patients (62.29 ± 10.27 vs. 67.37 ± 9.29 years, *p* < 0.001) and had a lower prevalence of diabetes (27.2% vs. 38.5%, *p* < 0.001). They also exhibited lower rates of chronic obstructive pulmonary disease (COPD) or asthma (7.9% vs. 12.4%, *p* = 0.020) and atrial fibrillation (1.5% vs. 4.4%, *p* = 0.014). Differences were also observed in New York Heart Association (NYHA) functional class distribution (*p* < 0.001), with TAG patients more frequently in lower classes, and in the extent of coronary artery disease (*p* < 0.001), with SAG patients more often presenting with three-vessel disease. Previous cardiac surgery was more common in the TAG group (2.7% vs. 1.0%, *p* = 0.011). After propensity score matching, all baseline characteristics were well balanced between groups, with no remaining statistically significant differences and all SMDs below 0.10, indicating successful matching and comparability of the two cohorts. Covariate balance before and after matching is illustrated in Figure 2.

### 3.2. Intraoperative Data

Intraoperative characteristics are shown in Table 2. In the unmatched cohort, TAG patients underwent OPCAB more frequently than SAG patients (60.4% vs. 45.1%, *p* < 0.001), received fewer grafts (2.55 ± 0.64 vs. 2.87 ± 0.68, *p* < 0.001), and had shorter CPB times (71 vs. 80 min, *p* = 0.011). After propensity matching, these differences were no longer significant, with OPCAB use, graft number, and other intraoperative parameters comparable between groups. The composition of the TAG cohort according to arterial conduit configuration is presented in Table 3.

### 3.3. In-Hospital Outcomes and Short-Term Mortality

Postoperative outcomes for both the unmatched and matched cohorts are summarised in Table 4. In the unmatched population, most in-hospital complications occurred at similar rates between SAG and TAG, with the exception of tracheostomy, which was significantly less common in the TAG group (0.6% vs. 2.7%, *p* = 0.020). No other postoperative outcomes—including reoperation for bleeding, stroke or TIA, deep sternal wound infection, renal replacement therapy, or 30-day mortality—showed statistically significant differences before matching. After propensity score matching, all short-term outcomes were comparable between groups, with no significant differences observed.

### 3.4. Survival Outcomes

Long-term follow-up differed significantly between groups in the unmatched cohort, with TAG patients having a longer mean follow-up duration than SAG patients (12.16 ± 7.7 vs. 10.13 ± 7.3 years, *p* < 0.001). After propensity matching, follow-up time was well balanced between groups (12.1 ± 7.6 vs. 11.7 ± 7.5 years, *p* = 0.544), ensuring comparability of survival estimates.

**Figure 3:**
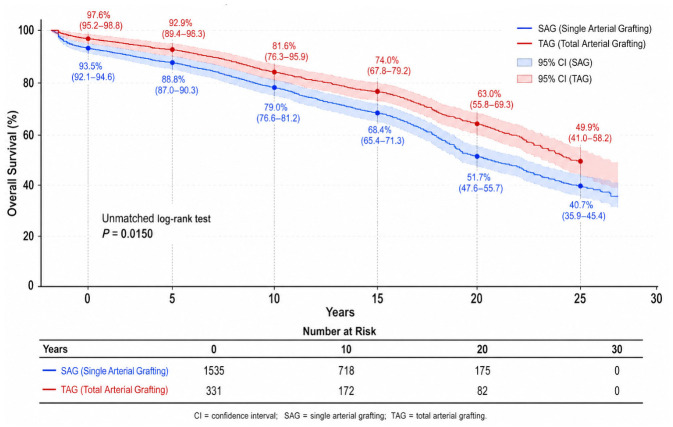
Kaplan–Meier estimates of long-term survival in the unmatched cohort according to conduit strategy.

Kaplan–Meier survival analyses for the unmatched cohort are presented in Table 5 and illustrated in Figure 3. TAG was associated with significantly improved long-term survival compared with SAG (log-rank *p* = 0.015). At 10 years, survival was 81.6% for TAG versus 79.0% for SAG, and this advantage persisted at 15 years (74.0% vs. 68.4%). The divergence in survival curves continued through 20 and 25 years, with TAG consistently demonstrating higher survival estimates.

**Figure 4:**
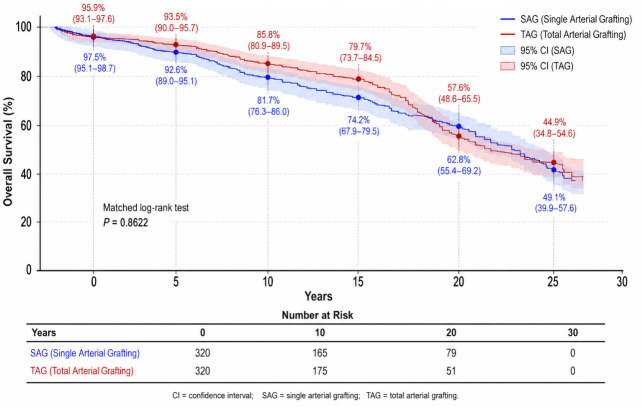
Kaplan–Meier estimates of long-term survival in the matched cohort according to conduit strategy.

In the matched cohort, survival outcomes are shown in Table 6 and depicted in Figure 4. Kaplan–Meier survival analysis in the propensity-matched cohort demonstrated no statistically significant difference in long-term survival between TAG and SAG groups (log-rank *p* = 0.862). Although numerical differences were observed at 10 and 15 years in favour of TAG, these did not reach statistical significance and were accompanied by overlapping confidence intervals throughout follow-up.

Beyond 15 years, survival estimates became progressively less precise due to declining numbers at risk. At 20 and 25 years of follow-up, the number of patients remaining under observation was substantially reduced, and therefore these late estimates should be interpreted with caution. The apparent separation and subsequent convergence of survival curves at these extended time points is considered exploratory and reflects increasing statistical uncertainty rather than a definitive difference in outcomes between groups.

Overall, the matched analysis demonstrates comparable long-term survival between TAG and SAG, with reliable estimates primarily supported in the early and mid-term follow-up period.

Overall, while unadjusted analyses suggested improved survival with TAG, this difference was not observed after propensity matching, and adjusted analyses demonstrated no statistically significant difference in long-term survival between groups.

### 3.5. Predictors of Long-Term Mortality

Univariable and multivariable Cox regression analyses are presented in Table 7. Independent predictors of long-term mortality included age (HR 1.045, *p* < 0.001), NYHA class, previous cardiac surgery (HR 0.501, *p* = 0.006), and OPCAB (HR 1.521, *p* < 0.001). Grafting strategy (TAG vs. SAG) was not independently associated with mortality (HR 1.005, *p* = 0.966). Off-pump coronary artery bypass grafting (OPCAB) was included in the multivariable model and remained independently associated with increased long-term mortality; however, baseline differences in operative strategy were substantially attenuated following propensity score matching.

### 3.6. Sensitivity Analyses for Calendar-Time Effects

To further evaluate the potential impact of temporal changes in surgical practice, additional sensitivity analyses incorporating calendar time were performed. Inclusion of year of surgery as a continuous covariate in the multivariable Cox model did not demonstrate a significant association with long-term mortality and did not materially alter the effect estimate for grafting strategy (TAG vs. SAG), which remained non-significant.

In era-stratified analyses, no significant association between conduit strategy and long-term survival was observed within the early (1996–2004), mid (2005–2014), or late (2015–2023) operative periods. Hazard ratios for TAG versus SAG remained consistently non-significant across all eras, with no evidence of temporal interaction.

These findings suggest that the neutral association between grafting strategy and long-term survival is robust and not confounded by temporal changes in surgical techniques, perioperative care, or secondary prevention.

## 4. Discussion

In this large retrospective cohort of patients with moderate LV dysfunction undergoing isolated CABG, TAG was not independently associated with improved long-term survival after adjustment, although unadjusted analyses suggested a numerical difference in favour of TAG. Propensity-matched analysis demonstrated that conduit strategy did not independently predict long-term mortality, which was instead driven by age, NYHA class, previous cardiac surgery, and OPCAB.

Importantly, interpretation of late survival beyond 15–20 years must be made cautiously, as the number of patients at risk declines substantially over time, resulting in wider confidence intervals and reduced statistical reliability.

In the present study, survival estimates at 20 and 25 years are based on a limited subset of the original matched cohort, and therefore should be considered exploratory rather than confirmatory. The observed late separation and subsequent crossing of Kaplan–Meier curves likely reflect stochastic variation due to small numbers rather than a true biological divergence between grafting strategies. Accordingly, no clinical conclusions should be drawn from very late survival behaviour.

The primary and most robust finding of this study remains the absence of a statistically significant difference in adjusted long-term survival between TAG and SAG, with outcomes driven predominantly by patient-related factors rather than conduit selection.

Our findings align with earlier work demonstrating the safety of arterial grafting in impaired LV function. Deng et al. reported low perioperative mortality (2.2%) and acceptable morbidity in patients undergoing TAG with two arterial conduits [1]. Similarly, Chung et al. showed that OPCAB with bilateral internal mammary arteries achieved favourable early and mid-term outcomes even in severe LV dysfunction [2]. More recently, a large binational registry analysis demonstrated that multiarterial grafting confers a consistent survival benefit across all LVEF strata, including moderate dysfunction [7]. Although numerical differences in survival were observed in favour of TAG, these were not statistically significant and were accompanied by overlapping confidence intervals. As such, the present findings do not confirm a survival advantage of TAG in this population.

Arterial conduits have well-described biological advantages, including improved long-term patency, resistance to atherosclerosis, and preserved endothelial function when compared with saphenous vein grafts [8,9]. However, these attributes have been demonstrated primarily in angiographic and observational studies, and in the present analysis, no direct assessment of graft patency, serial imaging, or cause-specific mortality was available. As such, these mechanistic pathways cannot be evaluated within the context of the current study.

Accordingly, any potential physiological advantages of TAG should be considered hypothesis-generating rather than explanatory of the observed clinical outcomes. While prior literature has suggested that arterial grafting may be associated with improved myocardial perfusion and ventricular recovery in selected populations, such effects cannot be inferred from the neutral adjusted survival findings demonstrated in this cohort.

The principal finding of this study remains the absence of a statistically significant difference in long-term survival between TAG and SAG after propensity score adjustment, and all mechanistic interpretations should therefore be interpreted in light of this neutral result.

In keeping with the neutral mechanistic interpretation of the present study, the absence of a statistically significant survival difference between TAG and SAG in the matched cohort suggests that patient-level factors may exert a stronger influence on long-term prognosis in moderate LV dysfunction [10]. Age, comorbidity burden, severity of heart failure symptoms, and operative factors such as the use of OPCAB were all independent predictors of mortality, whereas conduit strategy was not. This finding aligns with the notion that in patients with moderate LV impairment—who sit between low-risk and high-risk extremes—the competing risks associated with underlying disease may attenuate the measurable impact of conduit choice on survival [11,12].

An unexpected finding of the present analysis was the apparent protective association between previous cardiac surgery and long-term mortality. This observation should be interpreted with caution, as it is unlikely to represent a true causal relationship. Patients undergoing redo surgery may represent a highly selected subgroup with favourable physiological reserve who survived an initial cardiac intervention, thereby introducing survivorship and selection bias. In addition, the relatively small number of patients with prior cardiac surgery in the cohort may result in statistical instability of the hazard estimate. Accordingly, this finding is most appropriately considered hypothesis-generating and should not be overinterpreted.

OPCAB was independently associated with increased long-term mortality in the present study. This finding should be interpreted with caution, as it is unlikely to represent a direct causal effect and is not consistent with the majority of contemporary evidence [13,14]. Instead, it most likely reflects confounding by indication, with OPCAB being more frequently utilised in higher-risk patients and during earlier phases of the study period when surgical and perioperative practices differed. In addition, variations in surgeon experience and evolving case selection over the 27-year study period may have contributed to this signal. Importantly, baseline differences in the use of OPCAB between groups were substantially reduced following propensity score matching, suggesting improved comparability in the adjusted analysis. Taken together, this association is best interpreted as reflective of residual confounding rather than an intrinsic effect of surgical technique on long-term survival.

This study has several important strengths, but also notable limitations that should be considered when interpreting the findings. The retrospective analysis was based on a large, prospectively maintained institutional database, which enhances data completeness and reliability. The sizeable cohort of 1866 patients with moderate LV dysfunction, combined with follow-up extending to 25 years, provides one of the most comprehensive evaluations of conduit strategy in this specific population. The use of propensity-score matching to create well-balanced groups, along with multivariable Cox regression to identify independent predictors of mortality, strengthens internal validity and analytical rigour. Furthermore, the focus on a clearly defined LVEF range (30–49%) addresses an under-studied but clinically important subgroup.

Despite these strengths, several limitations warrant careful consideration. The retrospective, non-randomised design means that unmeasured confounding cannot be excluded, even after propensity matching. Conduit strategy was determined by the operating surgeon and may have been influenced by factors not fully captured within the dataset, including coronary anatomy, target-vessel quality, conduit availability, perceived operative risk, and surgeon-specific preferences or expertise. Furthermore, institutional adoption of arterial grafting evolved during the study period, with greater utilisation of total arterial revascularisation in later years. Although propensity score matching and additional sensitivity analyses were performed to minimise bias, residual confounding related to patient selection and conduit allocation cannot be completely excluded. A further important consideration is the potential for calendar-time confounding, given the 27-year study period during which substantial changes occurred in surgical techniques, conduit selection practices, perioperative care, and secondary prevention therapies. Earlier in the series, saphenous vein–based strategies were more commonly used, whereas later years saw increased adoption of arterial grafting and contemporary medical therapy. Although we adjusted for year of surgery within the propensity score model and performed additional sensitivity analyses stratified by operative era, residual confounding from unmeasured temporal factors cannot be fully excluded. These analyses, however, consistently demonstrated no independent association between conduit strategy and long-term mortality, supporting the robustness of the primary findings. Echocardiographic follow-up was not routinely available, precluding assessment of postoperative LV function, reverse remodelling, or graft-related perfusion changes. Although survival information was available for 100% of patients, cause-specific mortality and non-fatal cardiac events were not systematically captured, limiting the ability to explore mechanisms underlying observed survival patterns. Finally, this was a single-centre study conducted in a high-volume arterial grafting programme, which may limit generalisability to centres with different case-mix, surgical expertise, or conduit-use patterns.

These findings support the safety and feasibility of TAG in patients with moderate LV dysfunction, demonstrating comparable short- and long-term outcomes relative to SAG in adjusted analyses. In contrast to some prior observational and registry-based studies suggesting a survival benefit with multiarterial grafting [4,5,6,7], the present analysis did not demonstrate an independent association between conduit strategy and long-term mortality after propensity matching and risk adjustment.

While numerical differences in survival favouring TAG were observed, these did not reach statistical significance and should be interpreted cautiously. Accordingly, the current data do not provide evidence that TAG confers a survival advantage in this specific population, and the choice of conduit strategy should be considered within the broader context of patient characteristics, comorbidity burden, and surgical expertise.

Given that SAG remains widely used in routine practice, these results provide reassurance that TAG can be performed safely without increasing perioperative risk; however, they do not support a clear survival benefit over SAG in patients with moderate LV dysfunction. Prospective studies with standardised conduit selection, routine postoperative imaging, and long-term follow-up are needed to clarify the true survival impact of TAG in patients with moderate LV dysfunction, as such designs would allow more precise assessment of graft patency, myocardial recovery, and the durability of arterial revascularisation over time. Incorporating serial echocardiography or advanced imaging modalities such as CT angiography or cardiac MRI would enable evaluation of postoperative LV remodelling, graft flow characteristics, and the relationship between conduit choice and myocardial viability. Standardising surgical techniques—including conduit harvesting, graft configuration, and the use of on-pump versus off-pump strategies—would reduce procedural variability and allow more accurate attribution of outcomes to conduit strategy rather than technical differences. Long-term follow-up extending beyond a decade is essential, given the known late attrition of vein grafts and the delayed survival divergence often observed with arterial grafting. Although randomised controlled trials would provide the highest level of evidence, they remain challenging due to surgeon preference, patient selection, and ethical considerations; nevertheless, carefully designed pragmatic trials or multicentre prospective registries with mandatory data completeness could offer valuable insights. Together, these future research efforts would help determine whether TAG should be routinely recommended for patients with moderate LV dysfunction and guide evidence-based refinement of revascularisation strategies.

## 5. Conclusions

In patients with moderate left ventricular dysfunction undergoing isolated coronary artery bypass grafting, total arterial grafting is safe and feasible with comparable short-term outcomes. Although unadjusted analyses suggested improved long-term survival with total arterial grafting, this association was not observed after propensity matching or multivariable adjustment. Grafting strategy was not an independent predictor of mortality, suggesting that patient-related and clinical factors play a more dominant role in long-term outcomes.

## Figures and Tables

**Table 1:** Preoperative demographics.

	Unmatched (n = 1866)				Matched (n = 640)			
Variable	SAG (n = 1535)	TAG (n = 331)	*p* Value	SMD	SAG (n = 320)	TAG (n = 320)	*p* Value	SMD
Age	67.37 ± 9.29	62.29 ± 10.27	<0.001	0.52	62.68 ± 10.17	62.79 ± 9.56	0.882	0.01
Female gender	241 (15.7)	31 (9.4)	0.003	0.19	31 (9.7)	36 (11.3)	0.251	0.05
NYHA			<0.001	0.34			0.928	0.02
I	327 (21.3)	105 (31.7)			98 (30.6)	98 (30.6)		
II	633 (41.2)	143 (43.2)			139 (43.4)	133 (41.6)		
III	463 (30.2)	65 (19.6)			65 (20.3)	74 (23.1)		
IV	112 (7.3)	18 (5.4)			18 (5.6)	15 (4.7)		
Previous cardiac surgery	15 (1.0)	9 (2.7)	0.011	0.13	9 (2.8)	9 (2.8)	1.00	0.00
Hypercholesterolaemia	1143 (74.5)	259 (78.2)	0.148	0.09	249 (77.8)	257 (80.3)	0.437	0.06
Hypertension	1035 (67.4)	233 (70.4)	0.294	0.06	223 (69.7)	226 (70.6)	0.931	0.02
Smoking			0.957	0.01			0.991	0.01
Never	497 (32.4)	105 (31.7)			102 (31.9)	100 (31.3)		
Ex-smoker	855 (55.7)	185 (55.9)			180 (56.3)	176 (55.0)		
Current	183 (11.9)	41 (12.4)			38 (11.9)	44 (13.8)		
Renal impairment	63 (4.1)	14 (4.2)	0.917	0.01	12 (3.8)	16 (5.0)	0.440	0.06
Dialysis	34 (2.3)	8 (2.4)	0.399	0.01	6 (1.9)	8 (2.5)	0.505	0.04
COPD/Asthma	190 (12.4)	26 (7.9)	0.020	0.15	26 (8.1)	29 (9.1)	0.488	0.03
Diabetes	591 (38.5)	90 (27.2)	<0.001	0.25	89 (27.8)	93 (29.1)	0.662	0.03
Cerebrovascular disease	110 (7.2)	16 (4.8)	0.125	0.10	41 (6.2)	47 (7.1)	0.699	0.04
Peripheral vascular disease	206 (13.4)	34 (10.3)	0.121	0.10	34 (10.6)	28 (8.8)	0.328	0.06
AF	67 (4.4)	5 (1.5)	0.014	0.17	5 (1.6)	5 (1.6)	0.254	0.00
Extent of CAD			<0.001	0.32			0.961	0.00
1	34 (2.2)	14 (4.2)			13 (4.1)	11 (3.4)		
2	278 (18.1)	97 (29.3)			91 (28.4)	93 (29.1)		
3	1223 (79.7)	220 (66.5)			216 (67.5)	216 (67.5)		
LMS disease	398 (25.9)	73 (22.1)	0.141	0.09	70 (21.9)	74 (23.1)	0.509	0.03
Logistic EuroSCORE	4.53 ± 5.73	4.19 ± 5.22	0.311	0.06	4.22 ± 5.29	4.50 ± 6.23	0.544	0.05

Data n (%), mean ± standard deviation; AF = atrial fibrillation; CAD = coronary artery disease; COPD = chronic obstructive pulmonary disease; LMS = left main stem; NYHA = New York Heart Association; SAG = single arterial grafting; SMD = standardized mean difference; TAG = total arterial grafting.

**Table 2:** Intraoperative data.

	Unmatched (n = 1866)			Matched (n = 640)		
Variable	SAG (n = 1535)	TAG (n = 331)	*p* Value	SAG (n = 320)	TAG (n = 320)	*p* Value
OPCAB	693 (45.1)	200 (60.4)	<0.001	190 (59.4)	190 (59.4)	0.263
Number of grafts	2.87 ± 0.68	2.55 ± 0.64	<0.001	2.58 ± 0.63	2.54 ± 0.59	0.443
CPB time	80 [67–102]	71 [56–90]	0.011	83 [63–104]	72 [57–91]	0.013
Aortic cross clamp time	52 [38–64]	45 [31–59]	0.082	51 [38–65]	48 [35–60]	0.054
ICOR	1.19 ± 0.17	1.13 ± 0.11	0.056	1.18 ± 0.15	1.14 ± 0.10	0.674
LAD grafting	1535 (100)	331 (100)	1.00	320 (100)	320 (100)	1.00

Data n (%), mean ± standard deviation, median [interquartile range]; CPB = cardiopulmonary bypass; ICOR = index of completeness of revascularization; LAD = left anterior descending; OPCAB = off-pump coronary artery bypass; SAG = single arterial grafting; TAG = total arterial grafting.

**Table 3:** Distribution of arterial conduit configurations in patients undergoing total arterial grafting.

Conduit Configuration	Number of Patients (n = 331)	Percentage of TAG Cohort, %
BIMA only	192	58.0%
LIMA + radial artery only	72	21.8%
BIMA + radial artery only	67	20.2%

BIMA = bilateral internal mammary arteries; LIMA = left internal mammary artery; TAG = total arterial grafting.

**Table 4:** In-hospital outcomes and short-term mortality.

	Unmatched (n = 1866)			Matched (n = 640)		
Variable	SAG (n = 1535)	TAG (n = 331)	*p* Value	SAG (n = 320)	TAG (n = 320)	*p* Value
Reoperation	81 (5.3)	9 (2.7)	0.333	9 (2.8)	10 (3.1)	0.816
Tracheostomy	42 (2.7)	2 (0.6)	0.020	2 (0.6)	1 (0.3)	0.563
TIA/CVA	30 (2.0)	9 (2.7)	0.378	9 (2.8)	5 (1.6)	0.280
DSWI	24 (1.5)	8 (2.4)	0.368	8 (2.5)	4 (1.3)	0.244
RRT	71 (4.6)	13 (3.9)	0.407	13 (4.1)	12 (3.8)	0.838
Death at 30 days	45 (2.9)	6 (1.8)	0.257	6 (1.9)	9 (2.8)	0.433

Data n (%); CVA = cerebrovascular accident; DSWI = deep sternal wound infection; RRT = renal replacement therapy; SAG = single arterial grafting; TAG = total arterial grafting.

**Table 5:** Survival rates for unmatched cohort.

Time (Years)	Survival%	95% CI
**SAG**		
1	93.5	92.1–94.6
5	88.8	87.0–90.3
10	79.0	76.6–81.2
15	68.4	65.4–71.3
20	51.7	47.6–55.7
25	40.7	35.9–45.4
**TAG**		
1	97.6	95.2–98.8
5	92.9	89.4–95.3
10	81.6	76.3–85.9
15	74.0	67.8–79.2
20	63.0	55.8–69.3
25	49.9	41.0–58.2

Unmatched log rank *p* = 0.0150; CI = confidence interval; SAG = single arterial grafting; TAG = total arterial grafting.

**Table 6:** Survival rates for matched cohort.

Time (Years)	Survival %	95% CI
**SAG**		
1	97.5	95.1–98.7
5	92.6	89.0–95.1
10	81.7	76.3–86.0
15	74.2	67.9–79.5
20	62.8	55.4–69.2
25	49.1	39.9–57.6
**TAG**		
1	95.9	93.1–97.6
5	93.5	90.0–95.7
10	85.8	80.9–89.5
15	79.7	73.7–84.5
20	57.6	48.6–65.5
25	44.9	34.8–54.6

Matched log rank *p* = 0.8622; CI = confidence interval; SAG = single arterial grafting; TAG = total arterial grafting.

**Table 7:** Univariable & Multivariable Cox regression for long-term mortality.

Univariable Cox Regression					Multivariable Cox Regression			
Variable	HR	95% CI Lower	95% CI Upper	*p*-Value	HR	95% CI Lower	95% CI Upper	*p*-Value
Age	1.045	1.035	1.055	0.005	1.045	1.035	1.058	<0.001
Female gender	0.805	0.642	1.011	0.062	0.882	0.701	1.109	0.283
NYHA Class								
NYHA II	0.579	0.412	0.813	0.002	0.562	0.400	0.790	<0.001
NYHA III	0.624	0.450	0.866	0.005	0.610	0.440	0.846	0.003
NYHA IV	0.798	0.571	1.114	0.185				
Previous cardiac surgery	0.512	0.316	0.830	0.007	0.501	0.307	0.819	0.006
Hypercholesterolemia	1.057	0.880	1.270	0.551				
Diabetes	0.868	0.725	1.039	0.123				
Hypertension	0.992	0.835	1.179	0.928				
Smoking	1.151	0.844	1.568	0.375				
Renal impairement	0.518	0.340	0.787	0.002				
RRT	0.726	0.230	2.294	0.585				
Pulmonary disease	0.868	0.657	1.146	0.317				
Cerebrovascular disease	0.907	0.644	1.277	0.576				
Peripheral vascular disease	0.811	0.628	1.046	0.106				
Preoperative AF	0.861	0.551	1.346	0.511				
Extent of coronary diease	1.040	0.872	1.239	0.665				
LMS disease	1.090	0.878	1.352	0.434				
Index of revascularisation	1.124	0.817	1.546	0.473				
OPCAB	1.396	1.174	1.661	<0.001	1.521	1.271	1.821	<0.001
TAG	0.755	0.604	0.943	0.013	1.005	0.795	1.271	0.966

AF = atrial fibrillation; LMS = left main stem; NYHA = New York Heart Association; OPCAB = off-pump coronary artery bypass; RRT = renal replacement therapy; TAG = total arterial grafting.

## Data Availability

The data supporting the findings of this study are not publicly available due to GDPR and institutional data-governance restrictions. De-identified data may be made available upon reasonable request to the corresponding author, subject to appropriate approvals and compliance with data-protection regulations.
